# Facile Design of C-Doped g-C_3_N_4_/Ov-BiOBr Z-Scheme Heterostructure with High Photocatalytic Performance

**DOI:** 10.3390/nano16130796

**Published:** 2026-06-27

**Authors:** Bo Wu, Xiansheng Yu, Jianhua Li, Xuekun Jin, Fengjuan Chen, Haiming Duan, Biaobing Cao

**Affiliations:** Key Laboratory of Solid State Physics and Devices Autonomous Region, School of Materials Science & Engineering, Xinjiang University, Urumqi 830046, China; wb1104754697@163.com (B.W.); yuxs997@sina.com (X.Y.); 18640646865@163.com (J.L.); duan91870@sina.com (H.D.); bbcao@xju.edu.cn (B.C.)

**Keywords:** BiOBr, photocatalysis, heterojunction, oxygen vacancy, DFT theoretical calculation

## Abstract

Solar-driven photocatalysis has attracted increasing interest as an efficient and environmentally friendly approach for the mineralization of pollutants. In this work, carbon-doped g-C_3_N_4_/V_o_BiOBr composites rich in oxygen vacancy (denoted as CCN/V_o_BOB) were prepared by combining calcination with a solvothermal method, using glucose as the carbon source. The obtained composites were comprehensively characterized by XRD, TEM, and XPS to investigate their crystal structure, morphology, and surface chemical states, and their photocatalytic activity was evaluated through the degradation of organic pollutants. Among the prepared samples, 3.2 wt% CCN/V_o_BOB exhibited the best photocatalytic performance, reaching 98% degradation of Rhodamine B (RhB) and 95% degradation of Methylene Blue (MB) within 90 min, which was significantly superior to that of V_o_BOB and g-C_3_N_4_/V_o_BOB. This enhanced activity can be attributed mainly to the synergistic effects of oxygen vacancy, carbon doping, and heterojunction construction. Their combined action not only regulates the band structure of V_o_BOB effectively, but also greatly inhibits the recombination of photogenerated electron–hole pairs. These results were further supported by UV-Vis DRS and transient photocurrent measurements. Radical trapping experiments indicated that superoxide radicals (O_2_^−^) were the dominant active species during the reaction. In addition, density functional theory (DFT) calculations provided further evidence for the above conclusions. On the basis of both experimental observations and theoretical analysis, a reasonable photocatalytic reaction mechanism was proposed. This work offers a useful strategy for designing highly efficient photocatalysts through the synergistic integration of oxygen vacancy, nonmetal doping, and heterojunction engineering, and thus promotes the application of photocatalytic technology in pollutant degradation.

## 1. Introduction

With the rapid growth of the economy and continuous progress of society, the ecological environment on which human survival depends has been subjected to increasingly serious pollution and degradation [1,2,3,4,5]. Therefore, it is of great importance to develop environmental remediation technologies that are efficient, stable, and sustainable [6,7,8,9,10]. Photocatalytic technology has been widely used in pollutant treatment because of its advantages of low energy consumption, environmental friendliness, and low cost. As a result, a variety of photocatalysts have been developed, including TiO_2_, g-C_3_N_4_, and BiOX (X = Cl, Br, I) [11,12,13]. Among them, BiOBr has received considerable attention due to its distinctive layered structure, which is favorable for charge separation and offers abundant active sites. Nevertheless, its photocatalytic performance is still limited by the fast recombination of photogenerated electron–hole pairs. For this reason, extensive efforts have been devoted to the rational construction of BiOBr-based heterostructures with improved charge transfer behavior and enhanced photocatalytic efficiency [14,15,16,17].

A range of modification strategies has been explored, including non-metal doping, oxygen vacancy engineering, heterojunction construction, and crystal facet engineering. Among these approaches, oxygen vacancy engineering has been recognized as an effective way to improve photocatalytic activity [18,19,20,21]. For instance, Liu et al. reported that an oxygen-vacancy-rich BiVO_4_/V_o_BOB photocatalyst can significantly enhance the photocatalytic degradation ability of BiVO_4_/BiOBr through the combined effects of Fermi level regulation and oxygen defect engineering [22]. It has been demonstrated that the introduction of oxygen vacancy can alter the transfer pathway of photogenerated charge carriers, changing it from a traditional Type-II mechanism to a Z-scheme process. This is because oxygen vacancy can generate defect states near the conduction band and narrow the band gap by forming impurity bands, thereby strengthening visible-light absorption. In addition, surface oxygen vacancy can trap photogenerated electrons or holes, which helps suppress carrier recombination and promotes the transfer of trapped charges to adsorbed reactants. However, oxygen vacancy may be replenished during the photocatalytic process, resulting in a gradual decline in activity. Meanwhile, single-modification strategies still show limited ability in simultaneously improving light absorption, charge separation, and surface redox reactions. Recent studies have shown that the simultaneous construction of crystal defects and heterojunction interfaces can provide a more effective route for improving photocatalytic performance. Crystal defects, such as vacancy or dopant-induced defect sites, can regulate the electronic structure and provide additional active sites, while heterojunction interfaces can promote directional charge migration and suppress the recombination of photogenerated electron–hole pairs. Therefore, the rational integration of defect engineering and heterojunction construction is beneficial for prolonging the lifetime of photogenerated charge carriers and enhancing the visible-light response of photocatalysts [23,24,25].

Constructing heterojunctions between BiOBr and other semiconductors has been proven to be an effective way to improve photocatalytic performance. Such heterojunctions can modify the overall optical absorption behavior of the composite, promote interfacial charge transfer, and inhibit charge carrier recombination, while the intrinsic band gap of BiOBr itself remains unchanged [26,27,28,29,30]. For example, Liu, D.N. et al. showed that coupling g-C_3_N_4_ with stacked BiOBr nanosheets rich in oxygen vacancy represents an effective surface engineering strategy for improving photocatalytic activity [31]. As a two-dimensional layered semiconductor with unique electronic properties and good physicochemical stability, g-C_3_N_4_ is capable of directly utilizing visible light to degrade organic dyes [32,33]. Even so, the photogenerated electron–hole pairs in g-C_3_N_4_ are highly susceptible to recombination, which leads to low quantum efficiency and poor photocatalytic performance [34,35]. Therefore, suppressing carrier recombination in g-C_3_N_4_ has effectively become an important scientific issue that needs to be addressed. Non-metal doping has been widely investigated as a strategy for regulating the electronic structure of g-C_3_N_4_, but its effect on photocatalytic performance is not universally positive. The actual influence of doping depends strongly on the dopant species, doping concentration, bonding configuration, and the resulting structural distortion. For g-C_3_N_4_, the sp^2^-hybridized C/N units form a delocalized π-conjugated polymeric framework, which provides a structural basis for visible-light absorption and carrier migration. Appropriate carbon incorporation can tune the local electronic structure of the heptazine framework, slightly narrow the band gap, enhance π-electron delocalization, and facilitate the migration of photogenerated charge carriers along the conjugated network. However, excessive or uncontrolled doping may generate deep defect states or recombination centers, resulting in decreased photocatalytic activity [36,37]. Thus, non-metal doping is considered an important strategy for improving the photocatalytic activity of g-C_3_N_4_ [38,39]. Since non-metal doping can effectively regulate the electronic structure of g-C_3_N_4_ (CN) and suppress carrier recombination, carbon-doped g-C_3_N_4_ (CCN) was selected in this work to couple with oxygen-vacancy-rich BiOBr (V_o_BOB). The CCN/V_o_BOB composites were prepared by a calcination-assisted solvothermal method using glucose as the carbon source. Their structural and physicochemical properties were systematically investigated by a series of characterization techniques. The photocatalytic activity of CCN/V_o_BOB was evaluated through the degradation of Rhodamine B (RhB) and Methylene Blue (MB). In addition, by combining free radical trapping experiments, valence-band X-ray photoelectron spectroscopy (VB-XPS), and density functional theory (DFT) calculations, a possible photocatalytic reaction mechanism of the heterojunction system was proposed.

## 2. Materials and Methods

### 2.1. Materials

Bismuth nitrate pentahydrate (Bi(NO_3_)_3_·5H_2_O), melamine (C_3_H_6_N_6_), glucose, absolute ethanol (EtOH), potassium bromide (KBr), polyvinylpyrrolidone (PVP, MW ≈ 40,000), ethylene glycol (EG), Rhodamine B (RhB), methylene blue (MB), and deionized water (DI water).

### 2.2. Synthesis of C-Doping g-C_3_N_4_(CCN) and C-g-C_3_N_4_/Vo-BiOBr (CCN/V_o_BOB)

CCN was prepared by thermal polycondensation using glucose as the carbon source. Glucose was selected because it contains abundant hydroxyl groups, is inexpensive, and exhibits controllable carbonization behavior, which is beneficial for achieving uniform carbon doping. In a typical synthesis, 1.5 g of melamine and 0.18 g of glucose were thoroughly mixed and ground into a fine powder with an agate mortar. The obtained mixture was then placed in an alumina crucible and heated in a tube furnace under an air atmosphere to 550 °C at a ramp rate of 5 °C·min^−1^. After the calcination process, the product was allowed to cool naturally to room temperature, collected, and ground into fine powder. It was then washed several times with deionized water and absolute ethanol to remove residual impurities, followed by drying at 80 °C for 10 h to yield the final CCN sample. The temperature was raised to 550 °C at a heating rate of 5 °C·min^−1^. After naturally cooling down to room temperature, the resulting yellow product was collected and ground into a fine powder. For comparison, pristine g-C_3_N_4_ (CN) was prepared under the same calcination conditions as CCN, except that glucose was not added. The obtained product was denoted as CN.

The CCN/V_o_BOB hybrid composites were synthesized by a simple hydrothermal method. Briefly, 970 mg of Bi(NO_3_)_3_·5H_2_O, 240 mg of KBr, 800 mg of PVP, 10 mL of ethylene glycol (EG), and 50 mL of deionized water were added sequentially into a beaker under continuous stirring. In this process, EG acted as a mildly reducing solvent and provided a reaction environment favorable for the formation of oxygen-vacancy-related defects in BiOBr. PVP was mainly used as a complexing and structure-directing agent (Figure S7). It could coordinate with Bi^3+^ through its carbonyl groups, regulate the hydrolysis and growth of BiOBr nanosheets, and improve the dispersion and structural stability of the product. Different amounts of CCN (0.01, 0.015, 0.02, 0.025, 0.03, and 0.035 g) were then added separately into the above solution, and the mixtures were stirred for 2 h to ensure uniform dispersion. After that, each suspension was transferred into a 100 mL Teflon-lined stainless-steel autoclave and maintained at 160 °C for 12 h. Once the hydrothermal reaction was completed, the resulting precipitates were collected by centrifugation and washed three times with deionized water and absolute ethanol. Finally, the samples were dried at 60 °C for 10 h. According to the mass ratio of CCN, the obtained oxygen-vacancy-containing composites were labeled as 1.6 wt% CCN/V_o_BOB, 2.4 wt% CCN/V_o_BOB, 3.2 wt% CCN/V_o_BOB, 3.9 wt% CCN/V_o_BOB, 4.7 wt% CCN/V_o_BOB, and 5.4 wt% CCN/V_o_BOB, respectively.

### 2.3. Characterizations

The structural and physicochemical characteristics of the as-prepared samples were systematically examined by a series of analytical techniques. The crystal structures of the samples were analyzed by powder X-ray diffraction (XRD, Bruker D8 Advance, Bruker AXS GmbH, Karlsruhe, Germany) using Cu Kα radiation. Transmission electron microscopy (TEM, FEI Tecnai G2 F20 S-Twin, FEI Company, Hillsboro, OR, USA) was employed to observe the morphology and microstructural features of the materials. The chemical states and bonding environments of the elements were further investigated by X-ray photoelectron spectroscopy (XPS, Thermo Scientific ESCALAB 250Xi, Thermo Fisher Scientific, Waltham, MA, USA) with an Al Kα X-ray source. The optical absorption properties were measured by UV-vis diffuse reflectance spectroscopy (UV-vis DRS, Lambda 650 UV-vis spectrophotometer, PerkinElmer, Waltham, MA, USA) equipped with an integrating sphere, using BaSO_4_ as the reference standard with 100% reflectance. Electron paramagnetic resonance (EPR) spectroscopy was carried out on a JES FA200 spectrometer (JEOL, Akishima, Tokyo, Japan) to identify the existence of an oxygen vacancy. In addition, the photoelectrochemical properties of the samples were evaluated by photocurrent response, electrochemical impedance spectroscopy (EIS), and Mott–Schottky measurements on an electrochemical workstation (CHI 660E, Shanghai Chenhua, Shanghai, China).

### 2.4. Photocatalytic Activity Test

To evaluate the photocatalytic activity of the synthesized samples, Rhodamine B (RhB, 10 mg/L) and Methylene Blue (MB, 10 mg/L) were selected as model pollutants. The photocatalytic degradation experiments were performed using a PCX-50B Discover (Perfectlight, Beijing, China) multi-channel photochemical reaction system equipped with 5 W LED irradiation modules. In this work, the [white LED/420 nm LED] module was used as the irradiation source, with a typical irradiance of approximately 30 mW·cm^−2^ at the sample position. The reaction vessel was placed in the fixed irradiation position of the instrument to ensure consistent illumination during the photocatalytic tests. The procedure was as follows. First, 10 mg of the photocatalyst was dispersed in 50 mL of RhB or MB solution with a concentration of 10 mg/L. The suspension was then magnetically stirred in the dark for 30 min to reach adsorption–desorption equilibrium. After that, the LED lamp was turned on to start the reaction. A 4 mL aliquot was taken every 15 min and centrifuged to remove the catalyst. The absorbance of the solution at its characteristic wavelength was measured with a UV-vis spectrophotometer, and the remaining dye concentration was calculated accordingly.

To identify the main reactive species involved in the photocatalytic process, 5 mL of 0.1 mmol/L scavenger solution was added separately to the RhB solution before irradiation, including isopropanol (IPA), p-benzoquinone (BQ), and potassium iodide (KI). The photocatalytic degradation experiments were then carried out following the same procedure described above. By comparing the degradation efficiencies obtained in the presence of different scavengers, the key reactive species were determined.

### 2.5. Computational Details

**Figure 1:**
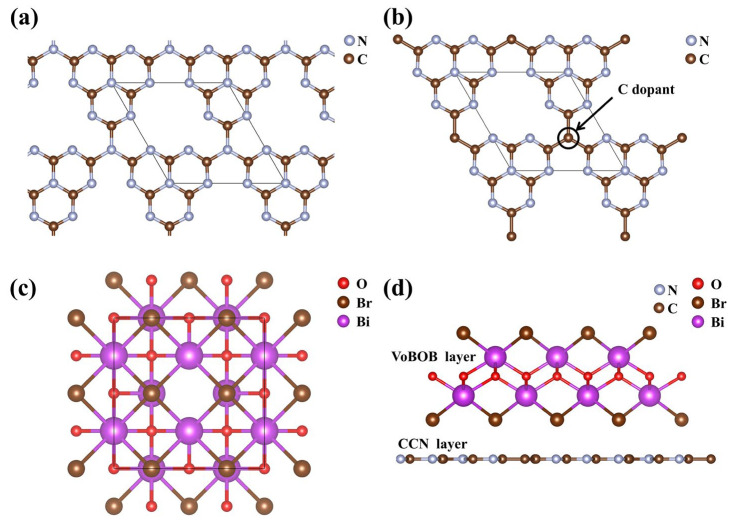
Atomic structural models of (**a**) CN, (**b**) CCN, (**c**) the V_o_BOB layer, and (**d**) the CCN/V_o_BOB heterojunction.

This study systematically calculated the electronic structures and work functions of CN, CCN, oxygen-vacancy-containing V_o_BOB, and the CCN/V_o_BOB heterojunction using the Vienna Ab initio Simulation Package (VASP, version 6.6.5.0) [40]. The atomic structural models used in the theoretical calculations are shown in Figure 1. The CN model was constructed based on heptazine-ring structural units. The CCN model was built by substituting one nitrogen atom in the CN heptazine unit with a carbon atom, and the corresponding doping site is marked in the figure to analyze the qualitative effect of carbon incorporation on the electronic structure of g-C_3_N_4_. The V_o_BOB model was constructed by removing one oxygen atom from the BiOBr surface to introduce an oxygen vacancy. The CCN/V_o_BOB heterojunction model was established through a layered stacking configuration, in which the CCN layer was placed on the surface of the V_o_BOB layer. The interfacial structure was then geometrically optimized to obtain a stable contact configuration. This heterojunction model was mainly used to investigate the interfacial electronic structure, work-function variation, and charge-transfer tendency after contact between CCN and V_o_BOB.

The exchange-correlation potential was described using the HSE06 hybrid functional. To ensure accurate convergence of the total energy calculations, the plane-wave cutoff energy was set to 500 eV. Geometry optimization was carried out in a stepwise manner. Specifically, three progressively refined optimization schemes were applied in order of increasing computational complexity. To satisfy the periodic boundary conditions, a 4 × 4 × 1 k-point mesh was used for both the CN and CCN models, a 4 × 4 × 1 k-point mesh was used for the V_o_BOB model, and a 2 × 3 ×1 k-point mesh was adopted for the CCN/V_o_BOB heterojunction model. To avoid artificial interlayer interactions, a vacuum layer of 15 Å was introduced along the direction perpendicular to the slab model, namely the z-axis.

## 3. Results

### Structure and Morphology Characterizations

**Figure 2:**
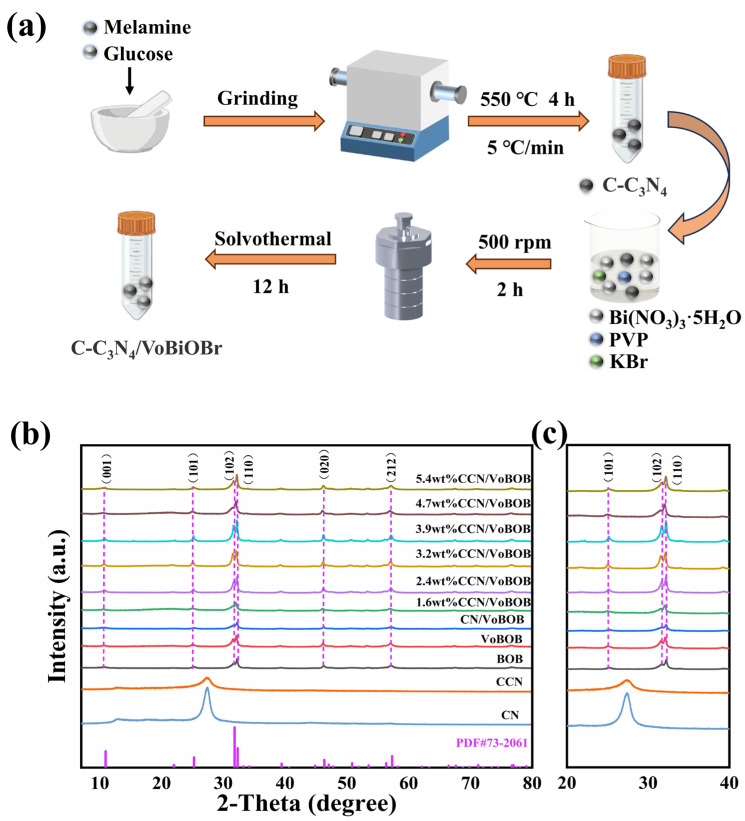
(**a**) The synthetic process of CCN/V_o_BOB heterojunction. In (**b**) and (**c**), the XRD patterns in different diffraction angle ranges from 5 to 80° and 20 to 40°.

As shown in Figure 2a, CCN was synthesized by thermal polycondensation using melamine as the precursor and glucose as the carbon source. For g-C_3_N_4_, the sp^2^-hybridized C/N units form an extended π-conjugated polymeric framework. Appropriate carbon incorporation can regulate the local electronic structure of the heptazine units, slightly narrow the band gap, and enhance π-electron delocalization, thereby promoting visible-light absorption and charge-carrier migration. After coupling with V_o_BOB, the regulated electronic structure of CCN, oxygen-vacancy-rich BiOBr, and the intimate heterojunction interface jointly contribute to the enhanced photocatalytic performance. A series of CCN/V_o_BOB composites was then prepared by a hydrothermal method. In this process, Bi(NO_3_)_3_·5H_2_O and KBr served as the bismuth and bromine sources, respectively. EG acted as a mildly reducing solvent and played the main role in creating an oxygen-vacancy-favorable environment for BiOBr. At 160 °C, EG can promote the partial removal of lattice oxygen from the BiOBr surface, resulting in oxygen-vacancy-related defects. Meanwhile, PVP mainly coordinates with Bi^3+^ ions through its carbonyl groups, slows down the hydrolysis process, and promotes the regulated growth of BiOBr nanosheets. Therefore, the oxygen vacancy in V_o_BOB is mainly associated with the EG-assisted solvothermal process, while PVP contributes mainly to morphology regulation and defect stabilization rather than directly generating oxygen vacancy. The crystal structures of the as-prepared products were characterized by X-ray diffraction (XRD), and the XRD patterns of all samples are shown in Figure 2b. CCN exhibits two distinct diffraction peaks at 13.2° and 27.5°, which correspond to the (100) and (002) crystal planes of CN, respectively (JCPDS 87-1526) [41]. The strongest peak at 27.5° is assigned to the interlayer stacking reflection of aromatic systems, whereas the weak peak at 13.2° is attributed to the in-plane structural packing of tri-s-triazine units. In addition, because carbon doping disrupts the long-range order of the CN framework, both diffraction peaks are very weak [42]. In the XRD patterns of the CCN/V_o_BOB composites, the diffraction peaks observed at 10.9°, 22.0°, 25.3°, 46.3°, and 57.3° can be indexed to the (001), (002), (101), (020), and (212) planes of tetragonal BiOBr (PDF No. 73-2061), respectively [43]. Figure 2c shows the locally enlarged XRD patterns. The diffraction peak corresponding to the (102) plane shifts toward a lower angle, and this shift becomes more pronounced with increasing CCN content. Such peak displacement indicates changes in the lattice spacing and local structure of BiOBr during the compositing process, which are associated with interfacial interactions in CCN/V_o_BOB, confirming the formation of a heterostructure between CCN and V_o_BOB [44,45].

**Figure 3:**
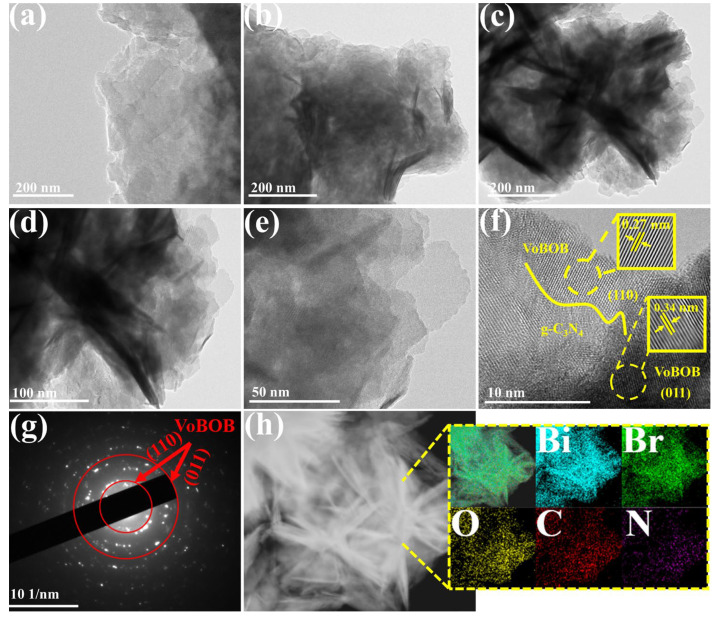
TEM images of (**a**) CCN, (**b**) V_o_BOB and (**c**–**e**) CCN/V_o_BOB. (**f**) HRTEM images of CCN/V_o_BOB. (**g**) SAED pattern and (**h**) elemental mappings of CCN/V_o_BOB.

The microstructure of CCN, V_o_BOB, and CCN/V_o_BOB was analyzed using transmission electron microscopy (TEM). As shown in Figure 3a, CCN exhibits a loosely stacked, porous, and two-dimensional wrinkled layered structure, consistent with the (002) crystal plane serving as the primary supporting plane [46]. V_o_BOB predominantly consists of vertically aligned nanosheets (Figure 3b). In the CCN/V_o_BOB composite (Figure 3c–e), V_o_BOB nanosheets are randomly distributed on the larger CCN layers, further confirming the successful synthesis of the heterostructure. High-resolution TEM (HRTEM) images (Figure 3f) reveal distinct lattice fringes corresponding to the (110) and (011) planes of V_o_BOB, coexisting with disordered lattice regions of CCN. Additionally, the selected-area electron diffraction (SAED) pattern of CCN/V_o_BOB (Figure 3g) shows polycrystalline diffraction rings, which can be assigned to the (110) and (011) planes of V_o_BOB. Elemental mapping results (Figure 3h) further demonstrate the uniform distribution of Br, Bi, C, N, and O throughout the composite, confirming the successful synthesis of CCN/V_o_BOB.

**Figure 4:**
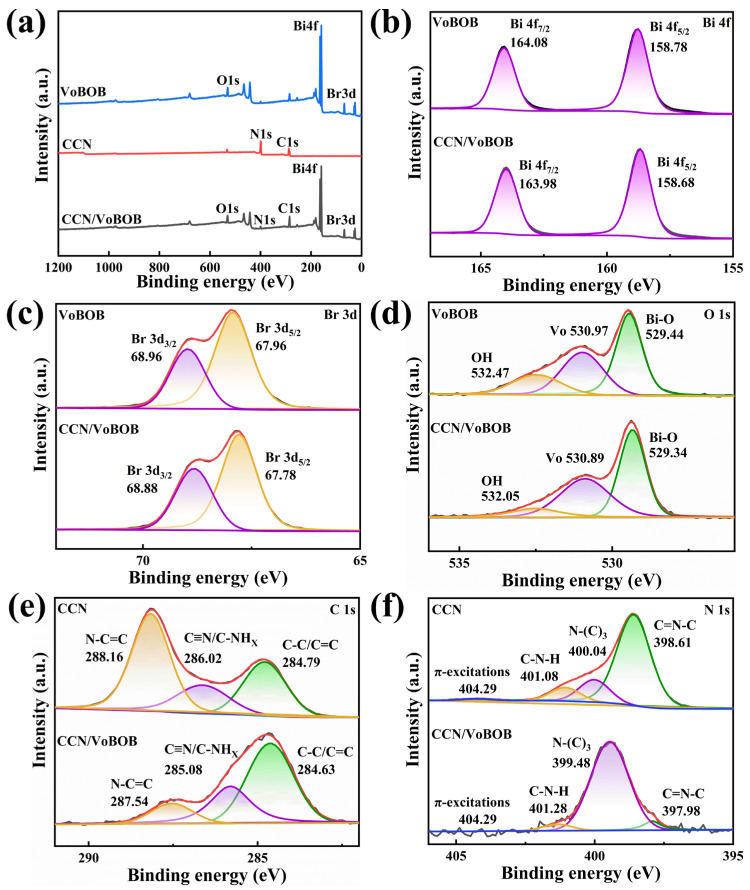
(**a**) XPS survey spectra of V_o_BOB, CCN and CCN/V_o_BOB; High-resolution XPS spectra of V_o_BOB, CCN and CCN/VoBOB, Bi 4f (**b**), Br 3d (**c**), O1s (**d**), C 1s (**e**) and N 1s (**f**) of samples.

X-ray photoelectron spectroscopy (XPS) was used to determine the elemental composition and chemical states of the photocatalysts. As shown in the survey spectrum in Figure 4a, Bi, O, Br, C, and N are present in CCN/V_o_BOB. For V_o_BOB, the Bi 4f spectrum (Figure 4b) shows two peaks at 158.78 eV and 164.08 eV, corresponding to Bi 4f_7/2_ and Bi 4f_5/2_, respectively, while the Br 3d spectrum (Figure 4c) exhibits peaks at 67.96 eV and 68.96 eV, assigned to Br 3d_5/2_ and Br 3d_3/2_, respectively. In CCN/V_o_BOB, both the Bi 4f and Br 3d peaks shift by about 0.2 eV toward lower binding energies, indicating strong electronic interaction at the heterojunction interface. The Bi/Br atomic ratio is close to the stoichiometric value (Bi/Br ≈ 0.88, Table S1), suggesting that the BiOBr lattice is largely retained. The O 1s spectrum (Figure 4d) can be fitted into three components: lattice oxygen at 529.44 eV, oxygen vacancy at 530.97 eV, and surface-adsorbed oxygen at 532.47 eV [47]. Quantitative analysis shows that oxygen-vacancy-related oxygen species are present in both V_o_BOB and CCN/V_o_BOB. The oxygen-vacancy-related component accounts for about 23% in V_o_BOB and about 27% in CCN/V_o_BOB, indicating that the EG-assisted solvothermal process is favorable for the generation of oxygen-vacancy-related defects in BiOBr. After coupling with CCN, these vacancy-related species are still preserved, suggesting that the defect-rich structure remains stable during the formation of the CCN/V_o_BOB heterojunction. The C 1s spectrum of CCN (Figure 4e) shows three peaks at 284.79 eV, 286.2 eV, and 288.16 eV, which are assigned to C-C/C=C, C≡C/C-NH_X_, and N-C=C, respectively. Quantitative XPS analysis shows that the C/N atomic ratio increases from 0.78 in CN to 1.06 in CCN, while the relative nitrogen content decreases after glucose-assisted calcination, suggesting the incorporation of additional carbon into the g-C_3_N_4_ framework. The N 1s spectrum (Figure 4f) reveals four nitrogen species in CCN, corresponding to C-N=C, N-(C)_3_, C-N-H, and the π-excitation-related peak, indicating that the typical carbon nitride structural units of g-C_3_N_4_ are retained after carbon incorporation. Compared with CN, the relative proportion of C-N=C decreases, whereas that of N-(C)_3_ increases, suggesting that carbon incorporation changes the local chemical environment of the g-C_3_N_4_ framework. In addition, the XRD peak weakening of CCN and the DFT model based on N-site substitution by C further support the formation of carbon-doped g-C_3_N_4_. In CCN/V_o_BOB, all N 1s peaks, except the π-excitation peak, shift toward lower binding energies, further indicating interfacial electronic interaction between CCN and V_o_BOB. This interaction is favorable for the interfacial migration of photogenerated charge carriers in the composite system [48]. To further verify the presence of oxygen vacancy, EPR measurements were carried out, as shown in Figure S1. Pristine BiOBr shows no obvious EPR signal, whereas CCN/V_o_BOB exhibits a distinct signal at g = 2.003, indicating the formation of stable unpaired-electron centers after composite formation and oxygen-vacancy regulation. Combined with the O 1s XPS results, where oxygen-vacancy-related peaks are observed in both V_o_BOB and CCN/V_o_BOB and the vacancy component is retained in CCN/V_o_BOB, this EPR signal can be mainly attributed to electron localization caused by oxygen-vacancy-related defects in V_o_BOB and their interfacial interaction with CCN. Taken together, the XPS fitting results, EPR response, and the changes in the optical and electronic properties support the formation of a closely contacted defect-rich CCN/V_o_BOB heterojunction.

**Figure 5:**
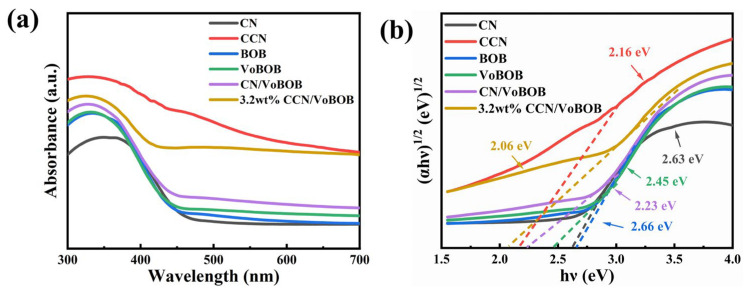
(**a**) UV-vis DRS, and (**b**) Tauc plots of BOB, V_o_BOB, CN, CCN, CN/BOB and 3.2 wt% CCN/V_o_BOB.

The optical properties of the samples were investigated by ultraviolet–visible diffuse reflectance spectroscopy (DRS). As shown in Figure 5a, CCN exhibits much stronger visible-light absorption in the range of 450–700 nm than CN, suggesting that carbon doping can effectively improve the visible-light utilization ability of CN. This result is also reflected in the apparent color change in the samples, with pristine CN showing a light-yellow color and CCN appearing gray-black. In a similar way, V_o_BOB shows significantly stronger visible-light absorption than BOB, indicating that the introduction of oxygen vacancy is beneficial for light harvesting. After CCN was coupled with V_o_BOB to form a heterojunction, the absorption edge of the CCN/V_o_BOB composite shifted further toward longer wavelengths, revealing a further enhancement in visible-light response. As shown in Figure S2a, the notably improved visible-light absorption of 3.2 wt% CCN/V_o_BOB (hereafter denoted as CCN/V_o_BOB) is mainly associated with the successful formation of the heterojunction structure. The optical band gaps of the samples were further estimated from Tauc plots according to the equation (αhν)^1/2^ = A(hν−E_g_), where α is the absorption coefficient, hν is the photon energy, A is a constant, and E_g_ represents the band gap energy [49,50]. The calculated band gap values for CN, CCN, BOB, V_o_BOB, CN/V_o_BOB, and 3.2 wt% CCN/V_o_BOB are 2.63 eV, 2.16 eV, 2.66 eV, 2.45 eV, 2.23 eV, and 2.06 eV, respectively (Figure 4b). The decrease in band gap from 2.63 eV for CN to 2.16 eV for CCN can be ascribed to the introduction of mid-gap states by carbon doping, which provide intermediate energy levels for sub-bandgap photon absorption and facilitate electron excitation under visible light [51,52]. Among all the samples, Figure S2b shows that 3.2 wt% CCN/V_o_BOB has the smallest band gap, indicating that the combined effects of oxygen vacancy, carbon doping, and heterojunction construction can effectively narrow the band gap and thereby improve photocatalytic performance [53,54].

**Figure 6:**
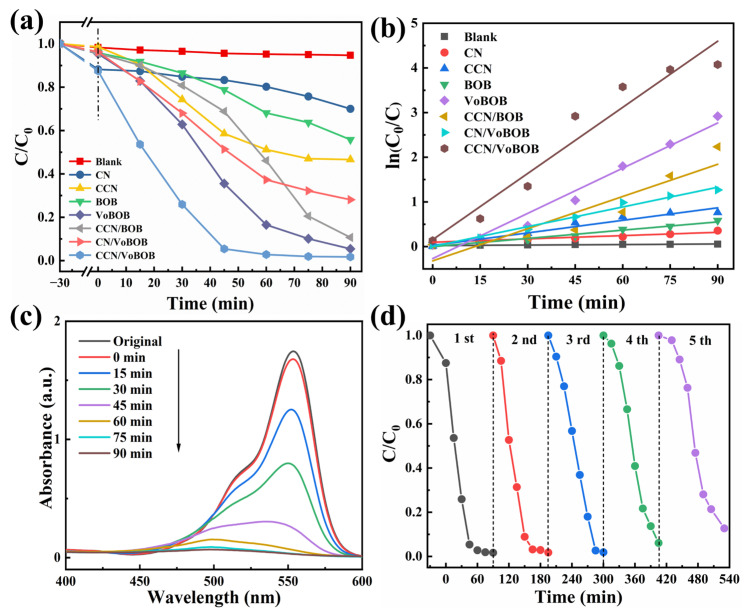
(**a**,**b**) Photocatalytic degradation activity of RhB under visible light for CN, CCN, BOB, V_o_BOB, CCN/BOB, CN/V_o_BOB, and CCN/V_o_BOB. (**c**) Temporal evolution of the characteristic absorption peaks of RhB in the presence of CCN/V_o_BOB. (**d**) Cyclic stability test of the CCN/V_o_BOB for RhB.

The photocatalytic performance of CCN/V_o_BOB was evaluated by degrading Rhodamine B (RhB, 10 mg/L) solution under LED. As shown in Figure 6a, compared with BOB, V_o_BOB exhibited higher degradation efficiency toward RhB in 90 min, demonstrating that the introduction of oxygen vacancy can enhance the photocatalytic activity. Similarly, CCN also displayed higher degradation performance compared to CN, indicating that carbon doping is helpful to improve performance. When CCN was combined with V_o_BOB (Figure S3), 3.2 wt% CCN/V_o_BOB exhibited the highest photocatalytic performance, achieving a RhB degradation rate of 98% within 90 min. Figure 6b analyzes the degradation process of RhB using the apparent pseudo-first-order kinetic plot of ln(C_0_/C) versus irradiation time. The rate constant k was obtained from the slope of the linear fitting curve and used to compare the photocatalytic degradation rates of different samples under the same experimental conditions. The rate constant k for CCN/V_o_BOB is 0.04936 min^−1^, which is 20.1, 5.3, 8.0, 1.5, 2.1, and 3.4 times higher than those of CN (0.00246 min^−1^), CCN (0.00936 min^−1^), BOB (0.00616 min^−1^), V_o_BOB (0.03372 min^−1^), CCN/BOB (0.02403 min^−1^), and CN/V_o_BOB (0.01471 min^−1^), respectively, indicating a synergistic effect between CCN and V_o_BOB. This synergy arises from carbon doping narrowing the bandgap and suppressing recombination, oxygen vacancy providing active sites and extending light absorption, and the heterojunction enabling efficient Z-scheme charge transfer that preserves strong redox potential. As seen in Figure 6c, the characteristic UV-vis absorption peak of RhB gradually decreases during irradiation and is almost completely eliminated within 90 min. Furthermore, cycling tests (Figure 6d) reveal only a slight decline in the degradation efficiency of CCN/V_o_BOB after five cycles, with degradation remaining above 92%, confirming its excellent stability. To further verify the structural stability of the heterojunction interface after photocatalytic reactions, TEM analysis was performed on the 3.2 wt% CCN/V_o_BOB catalyst collected after five cycling runs (Figure S4) [55,56]. The TEM images reveal that the composite retains its original morphology, with V_o_BOB nanosheets still uniformly distributed on the CCN layers. No significant aggregation or structural degradation is observed, indicating that the heterojunction interface remains intact during the photocatalytic process. This post-reaction characterization confirms that the catalyst possesses not only excellent activity but also robust structural stability.

**Figure 7:**
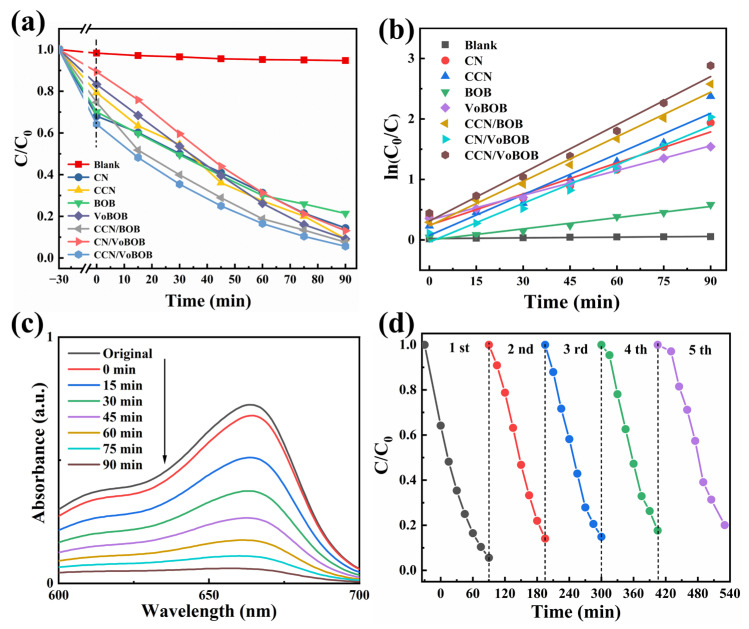
(**a**,**b**) Photocatalytic degradation activity of MB under visible light for CN, CCN, BOB, V_o_BOB, CCN/BOB, CN/V_o_BOB, and CCN/V_o_BOB. (**c**) Temporal evolution of the characteristic absorption peaks of MB in the presence of CCN/V_o_BOB. (**d**) Cyclic stability test of the CCN/V_o_BOB for MB.

The photocatalytic performance of CCN/V_o_BOB was also evaluated by degrading Methylene Blue (MB, 10 mg/L) solution under LED. As shown in Figure 7a, V_o_BOB demonstrated higher degradation capability for MB than BOB within 90 min, confirming that the introduction of oxygen vacancy is beneficial for improving the photocatalytic activity. Similarly, CCN exhibited higher degradation efficiency relative to CN, indicating that carbon doping can enhance the performance. When CCN was combined with V_o_BOB (Figure S5), 3.2 wt% CCN/V_o_BOB exhibited the highest photocatalytic performance (95%). Figure 7b analyzes the degradation process of MB using the apparent pseudo-first-order kinetic plot of ln(C_0_/C) versus irradiation time. The rate constant k was obtained from the slope of the linear fitting curve and used to compare the photocatalytic degradation rates of different samples under the same experimental conditions. The rate constant k for CCN/V_o_BOB is 0.02654 min^−1^, which is 1.55, 1.18, 4.31, 1.95, 1.08, and 1.25 times higher than those of CN (0.017124 min^−1^), CCN (0.022409 min^−1^), BOB (0.006164 min^−1^), V_o_BOB (0.013636 min^−1^), CCN/BOB (0.024545 min^−1^), and CN/V_o_BOB (0.021310 min^−1^), respectively, indicating a synergistic enhancement effect between CCN and V_o_BOB. This enhancement results from the triple synergy of carbon doping, oxygen vacancy, and heterojunction formation. In Figure 7c, the intensity of the characteristic UV-Vis absorption peak of MB gradually decreases over time and nearly disappears after 90 min. Furthermore, cycling tests (Figure 7d) show a minimal loss in the degradation performance of CCN/V_o_BOB after five cycles, demonstrating its good stability.

**Figure 8:**
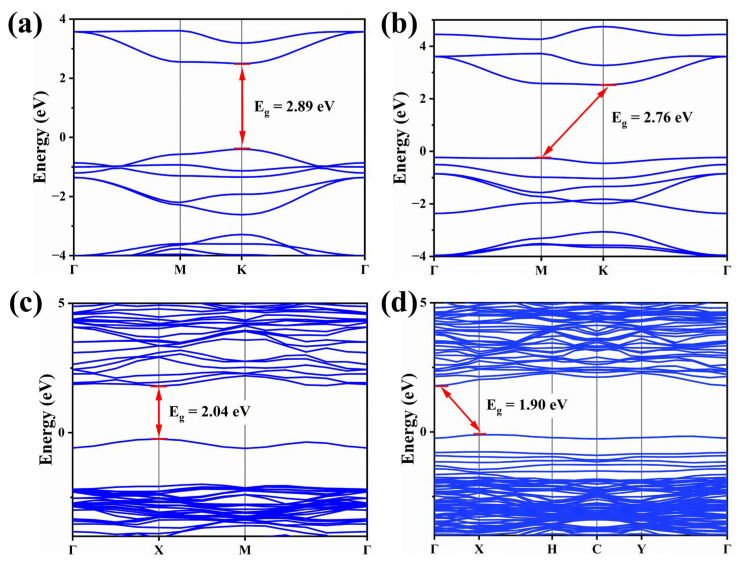
The band structures of (**a**) CN, (**b**) CCN, (**c**) V_o_BOB and (**d**) CCN/V_o_BOB.

To clarify how carbon doping affects the band structure of the material, systematic first-principles calculations based on density functional theory (DFT) were performed using the atomic models shown in Figure 1 [57,58]. The calculated band structures of CN, CCN, V_o_BOB, and CCN/V_o_BOB are presented in Figure 8. Both CCN and the CCN/V_o_BOB heterojunction show indirect-band-gap semiconductor characteristics, with calculated band gaps of 2.76 eV and 2.04 eV, respectively. Compared with the theoretical band gap of pristine CN, carbon doping leads to a narrower band gap. This narrowing can be attributed to the substitution of nitrogen atoms, which introduces electron-deficient states and mid-gap levels, thereby modifying the electronic structure. Such changes are beneficial for the separation and migration of photogenerated electron–hole pairs and thus improve the photocatalytic performance. In addition, the further reduced band gap of CCN/V_o_BOB indicates that carbon doping, oxygen vacancy, and heterointerface construction can jointly regulate the electronic structure of the system, which agrees well with the enhanced visible-light response of CCN/V_o_BOB observed experimentally.

**Figure 9:**
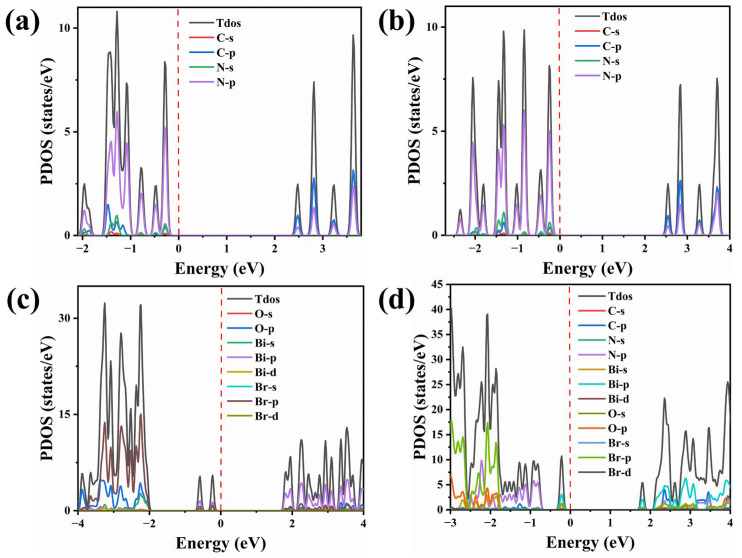
The DOS structures of (**a**) CN, (**b**) CCN, (**c**) V_o_BOB and (**d**) CCN/V_o_BOB.

To examine the electronic structure and the underlying modulation mechanism, density of states (DOS) and projected density of states (PDOS) calculations were performed [59,60]. As shown in Figure 9, the band structures and the corresponding DOS of CN, CCN, V_o_BOB, and the CCN/V_o_BOB heterojunction were obtained from first-principles calculations. The DOS provides a clear description of the band gap and electronic states, while the PDOS further identifies the atomic and orbital contributions to the electronic bands. The results show that, near the valence band maximum of CCN, the electronic states mainly originate from N 2p orbitals, whereas the conduction band minimum is composed of both N 2p and C 2p orbitals. For the CCN/V_o_BOB heterojunction, the valence band edge is mainly derived from the hybridization of Br 4p and N 2p orbitals, while the conduction band edge is dominated by Bi 6p, Br 4p, and C 2p orbitals. These results indicate a certain degree of electronic-state coupling between Br 4p orbitals and C 2p/N 2p orbitals at the heterojunction interface, which is favorable for interfacial charge transfer. In addition, the comparison of PDOS results shows an overlap between the density of states of CCN near the Fermi level and that of V_o_BOB at the corresponding energy range. This suggests that electronic channels favorable for carrier migration may form at the interface after contact between the two phases. Such an electronic configuration provides a suitable pathway for photogenerated electrons to transfer from CCN to V_o_BOB. This analysis at the electronic-structure level helps explain the enhanced photocatalytic performance of the heterojunction system.

**Figure 10:**
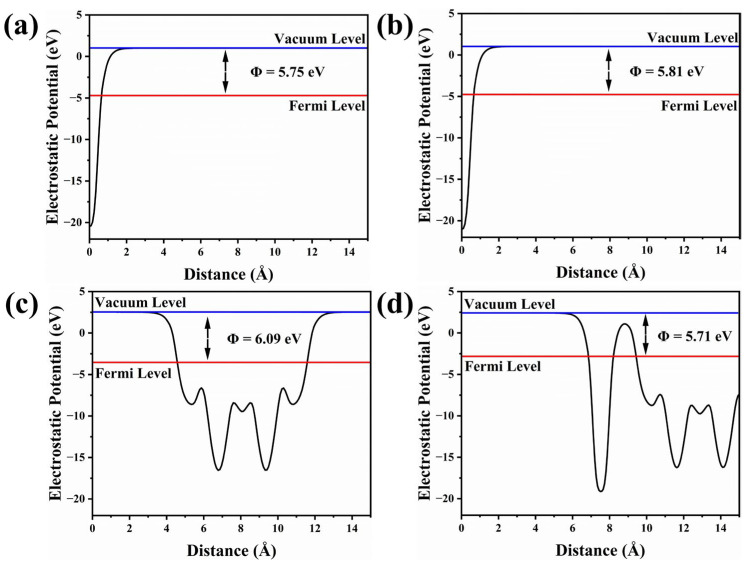
The work function structures of (**a**) CN, (**b**) CCN, (**c**) V_o_BOB and (**d**) CCN/V_o_BOB.

The work functions (Φ) of the individual components and the heterojunction were obtained from the plane-averaged electrostatic potential. As shown in Figure 10, the calculated work functions of CN, CCN, V_o_BOB, and the CCN/V_o_BOB heterojunction are 5.75, 5.81, 6.09, and 5.71 eV, respectively. Compared with CN, carbon doping slightly increases the work function of CCN. Nevertheless, a work-function difference of about 0.28 eV remains between CCN and V_o_BOB, with V_o_BOB showing the higher value. This indicates that, upon contact, electrons tend to transfer from CCN to V_o_BOB until the Fermi levels reach equilibrium, leading to the formation of an internal electric field directed from CCN to V_o_BOB. This internal electric field is expected to promote the directional migration of photogenerated carriers at the interface. Specifically, photogenerated electrons in the conduction band of V_o_BOB can migrate toward the interface and recombine with holes in the valence band of CCN. As a result, strongly reducing electrons are retained in the conduction band of CCN, while strongly oxidizing holes remain in the valence band of V_o_BOB. The work function of the CCN/V_o_BOB heterojunction decreases to the lowest value of 5.71 eV, suggesting that the heterostructure effectively lowers the overall electron-emission barrier of the system. From the viewpoint of interfacial electronic-structure regulation, the built-in electric field arising from work-function matching enables more efficient charge separation in the CCN/V_o_BOB heterojunction. Meanwhile, the lower surface work function is favorable for the participation of photogenerated electrons in surface reduction reactions, thereby contributing to the enhanced photocatalytic performance.

**Figure 11:**
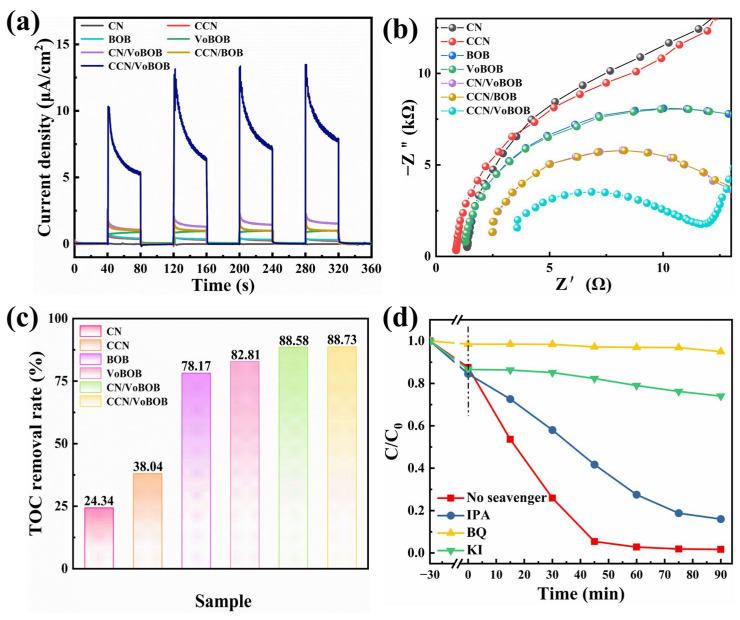
(**a**) Transient photocurrent responses. (**b**) EIS Nyquist plots. (**c**) TOC removal rate of the samples. (**d**) Degradation activity of CCN/V_o_BOB to RhB containing different scavengers.

To elucidate the separation and migration behavior of carriers, systematic characterization was performed using transient photocurrent response and electrochemical impedance spectroscopy (EIS) [61,62]. As shown in Figure 11a, the photocurrent density of CCN is higher than that of CN, indicating that carbon doping effectively enhances the migration of carriers, thereby improving its photocatalytic capability. Similarly, the photocurrent response of V_o_BOB is stronger than that of BOB, confirming that introducing an oxygen vacancy can improve the fast transport of carriers. Notably, the CCN/V_o_BOB hybrid demonstrates a stronger photocurrent response compared to V_o_BOB and CN/V_o_BOB. This enhancement arises from the synergistic contributions of each component: carbon doping narrows the bandgap of CN, enhancing visible-light absorption and generating more charge carriers; oxygen vacancy introduce defect states that extend light absorption, trap electrons to suppress recombination, and serve as active sites for interfacial charge transfer; and the heterojunction enables efficient spatial charge separation via a Z-scheme pathway, with the work function difference creating an internal electric field that drives directional carrier migration across the large 2D/2D contact interface. The electrochemical impedance spectroscopy (EIS) results (Figure 11b) further support the above conclusions [63,64]. The CCN exhibits a smaller arc radius than CN, and V_o_BOB a smaller one than BOB. CCN/V_o_BOB displays the smallest arc radius, indicating the lowest interfacial charge transfer resistance and the highest charge separation efficiency. This trend is highly consistent with the photocurrent response results, confirming that the combination of carbon doping, oxygen vacancy, and heterojunction construction can significantly enhance the separation efficiency of carriers, thereby optimizing the photocatalytic performance. To evaluate the deep mineralization capability, total organic carbon (TOC) analysis was conducted. In Figure 11c, CCN/V_o_BOB exhibits the highest mineralization rate (88.73% within 90 min), compared to 78% for pristine BiOBr, 82% for V_o_BOB, and 24% for CCN alone. The difference between degradation efficiency (98%) and TOC removal (88.73%) suggests that while most RhB molecules are mineralized to CO_2_ and H_2_O, a small fraction of intermediate products may persist and require longer irradiation for complete mineralization. To identify the key active species, radical trapping experiments were performed with different scavengers. Figure 11d presents the degradation kinetics of RhB over 3.2 wt% CCN/V_o_BOB in the presence of BQ (O_2_^−^ scavenger), IPA (·OH scavenger), and EDTA-2Na (h^+^ scavenger), and the corresponding pseudo-first-order rate constants (k) are compared in Figure S6. Upon addition of BQ, the rate constant k shows the most significant decrease, confirming that·O_2_^−^ plays the dominant role. In contrast, IPA and EDTA-2Na lead to relatively moderate reductions in k, indicating that OH and h^+^ are secondary active species. This bimodal active species distribution (dominant O_2_^−^ with significant OH contribution) is characteristic of a Z-scheme mechanism, where strongly reducing electrons in the CB of CCN (−0.26 eV) and strongly oxidizing holes in the VB of V_o_BOB (1.60 eV) are both preserved. If a Type-II heterojunction were formed, electrons would accumulate in the CB of V_o_BOB (−0.85 eV, still capable of O_2_^−^ generation), but holes would accumulate in the CB of CCN (1.90 eV), which has insufficient potential for OH generation (requires > 2.40 eV)—thereby contradicting the observed OH contribution. Thus, the trapping results, together with the band structure analysis and work function calculations, support a Z-scheme charge transfer pathway.

**Figure 12:**
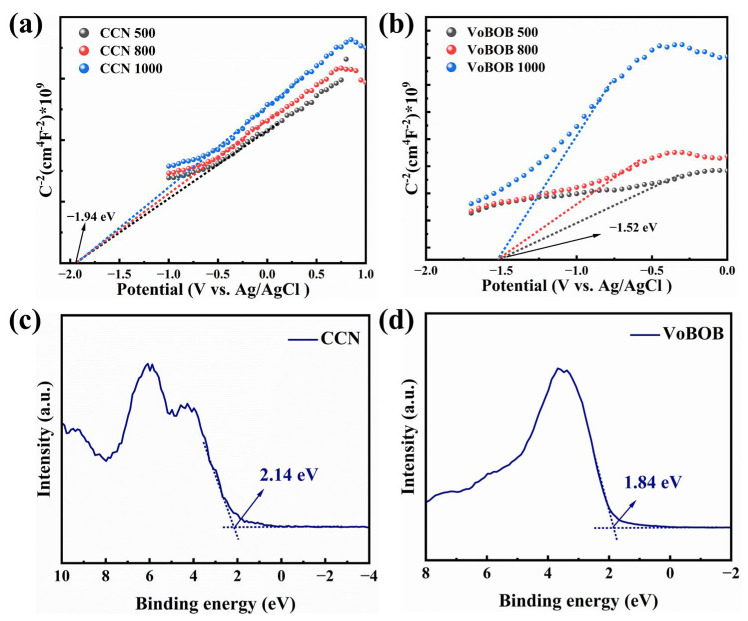
Mott–Schottky spectra of CCN (**a**) and V_o_BOB (**b**) XPS valence band spectra of CCN (**c**) and V_o_BOB (**d**).

The electronic band structures of the CCN and V_o_BOB were systematically investigated using Mott–Schottky (M-S) measurements and valence band X-ray photoelectron spectroscopy (VB-XPS) [65,66]. The M-S results (Figure 12a,b) show that they exhibit positive slopes in their plots, indicating the n-type semiconductor characteristics. The flat band potentials versus Ag/AgCl were determined to be −1.94 eV for CCN and −1.52 eV for V_o_BOB. Furthermore, the valence band maximum (VBM) positions obtained from VB-XPS (Figure 12c,d) were 2.14 eV for CCN and 1.84 eV for V_o_BOB. After calibration, the valence band potentials relative to the normal hydrogen electrode (NHE) were calculated to be 1.90 eV for CCN and 1.60 eV for V_o_BOB. The optical band gaps of CCN and V_o_BOB were found to be 2.16 eV and 2.45 eV, respectively (Figure 4). Based on these results, the conduction band minimum (CBM) potentials relative to NHE were derived as −0.26 eV for CCN and −0.85 eV for V_o_BOB. If a Type-II heterojunction were formed, electrons would accumulate in the CB of V_o_BOB (−0.85 eV) and holes in the VB of CCN (1.90 eV). While the CB potential of V_o_BOB is sufficient for O_2_^−^ generation, the VB potential of CCN is insufficient for OH generation. However, trapping experiments show a significant OH contribution, which cannot be explained by a Type-II mechanism, but it is consistent with a Z-scheme where holes are retained in the VB of V_o_BOB.

**Figure 13:**
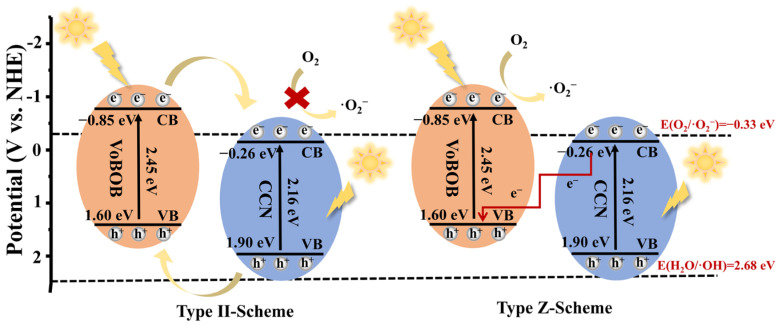
Photocatalytic mechanism of CCN/V_o_BOB.

Based on the results obtained from ultraviolet-visible diffuse reflectance spectroscopy (UV-vis DRS) and valence band X-ray photoelectron spectroscopy (VB-XPS), the photocatalytic mechanism of the CCN/V_o_BOB heterojunction was thoroughly investigated [67,68]. As illustrated in Figure 13, if the photocatalyst followed a Type-II heterojunction mechanism, photogenerated electrons would migrate from V_o_BOB, which has a more negative CB potential, to CCN with a more positive CB potential. Meanwhile, photogenerated holes would transfer from CCN, which possesses a more positive VB potential, to V_o_BOB. However, the conduction band potential (ECB) of CCN is lower than the redox potential of O_2_/O_2_^−^, so it cannot reduce dissolved oxygen to form·O_2_^−^. At the same time, the valence band potential (EVB) of V_o_BOB is also lower than the oxidation potential of·OH/H_2_O, meaning it is unable to oxidize H_2_O to generate OH. The above band structure analysis indicates that, under this hypothetical Type-II heterojunction model, neither O_2_^−^ nor OH reactive species can be produced. This conclusion contradicts the results obtained from the free radical trapping experiments, in which both species were observed to contribute significantly to the degradation process. Therefore, the CCN/V_o_BOB composite is more reasonably described by an interfacial Z-scheme-like charge-transfer pathway. In this process, photogenerated electrons in the CB of CCN tend to migrate toward the interface and recombine with holes in the VB of V_o_BOB. Oxygen-vacancy-related defect states may also participate in this interfacial recombination process and provide additional channels for charge redistribution. As a result, electrons with stronger reduction ability are retained in the CB of V_o_BOB, while holes with stronger oxidation ability remain in the VB of CCN. The retained electrons can reduce dissolved O_2_ to O_2_^−^, while the retained holes participate in oxidation reactions. This interpretation is supported by the radical trapping experiments, band-position analysis, and work-function results. Therefore, the enhanced photocatalytic activity of CCN/V_o_BOB can be attributed to the defect-mediated interfacial Z-scheme-like charge-transfer pathway, which promotes charge separation while maintaining relatively strong redox ability [69].

## 4. Conclusions

This work successfully prepared CCN/V_o_BOB photocatalysts through a two-step method consisting of calcination and subsequent solvothermal treatment. TEM observations showed that CCN possesses a porous nanosheet-like structure, while in the CCN/V_o_BOB composite, smaller V_o_BOB nanosheets are evenly distributed on the larger CCN sheets, forming close interfacial contact between the two components. Among the prepared samples, the catalyst with a CCN content of 3.2 wt% (denoted as 3.2 wt% CCN/V_o_BOB) exhibited the best photocatalytic performance, reaching degradation efficiencies of 98% for RhB and 95% for MB within 90 min under visible-light irradiation from an LED lamp. The improved photocatalytic activity can mainly be attributed to the synergistic effects of oxygen vacancy, carbon doping, and the incorporation of g-C_3_N_4_. DFT results further demonstrated that both oxygen vacancy and carbon doping can effectively reduce the band gap of CN, and that the subsequent formation of a heterojunction with BiOBr leads to an additional narrowing of the band gap, thereby strengthening light absorption and enhancing redox ability. In addition, the photocatalytic mechanism of CCN/V_o_BOB was systematically explored through radical trapping experiments, VB-XPS analysis, and UV-vis spectroscopy, which confirmed the formation of a Z-scheme heterojunction system. Such a mechanism promotes the efficient spatial separation of photogenerated electrons and holes at the interface, while at the same time maintaining strong oxidation and reduction capacities at the active sites. Overall, this study provides useful theoretical and experimental support for the development of highly efficient BiOBr-based photocatalysts.

## Data Availability

The original contributions presented in this study are included in the article. Further inquiries can be directed to the corresponding author.

## Supplementary Materials

The following supporting information can be downloaded at https://www.mdpi.com/article/10.3390/nano16130796/s1. Figure S1 presents EPR spectra of pristine BiOBr, V_o_BOB, and 3.2 wt% CCN/V_o_BOB. Figure S2 presents UV-vis DRS spectra and Tauc plots of CCN/V_o_BOB samples with different CCN contents (1.6 wt%, 2.4 wt%, 3.2 wt%, 3.9 wt%, 4.7 wt%, and 5.4 wt%). Figure S3 displays the photocatalytic degradation activity of RhB under visible light irradiation for the corresponding samples. Figure S4 displays TEM images of the 3.2 wt% CCN/V_o_BOB composite before and after five cycling runs. Figure S5 shows the photocatalytic degradation activity of MB under visible light irradiation for each CCN/V_o_BOB sample. Figure S6 presents the degradation kinetics of RhB over 3.2 wt% CCN/V_o_BOB in the presence of different scavengers. Figure S7. The O 1s XPS spectrum of V_o_BOB synthesized without PVP. Table S1 presents the surface elemental compositions of CN, CCN, BOB, V_o_BOB, and 3.2 wt% CCN/V_o_BOB determined by XPS.
