# Supplementary material for: Facile Design of C-Doped g-C3N4/Ov-BiOBr Z-Scheme Heterostructure with High Photocatalytic Performance
Source: Nanomaterials (Basel). 2026 Jun 27;16(13):796. doi: 10.3390/nano16130796 (PMC13363057; doi:10.3390/nano16130796)
Supplement: Supplementary file 1 [file nanomaterials-16-00796-s001.zip › nanomaterials-4402987-supplementary.pdf]

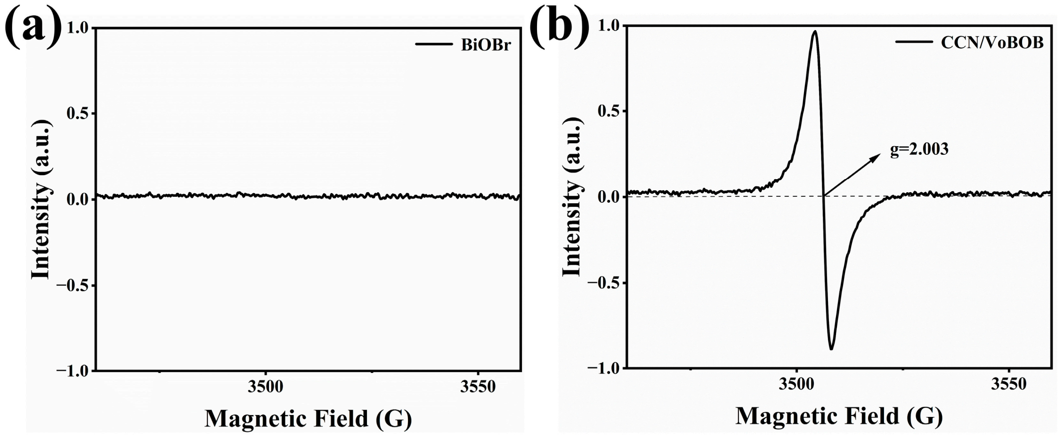

Figure S1. Room-temperature EPR spectra of (a) pristine BiOBr and (b) 3.2 wt% CCN/V$_o$BOB.

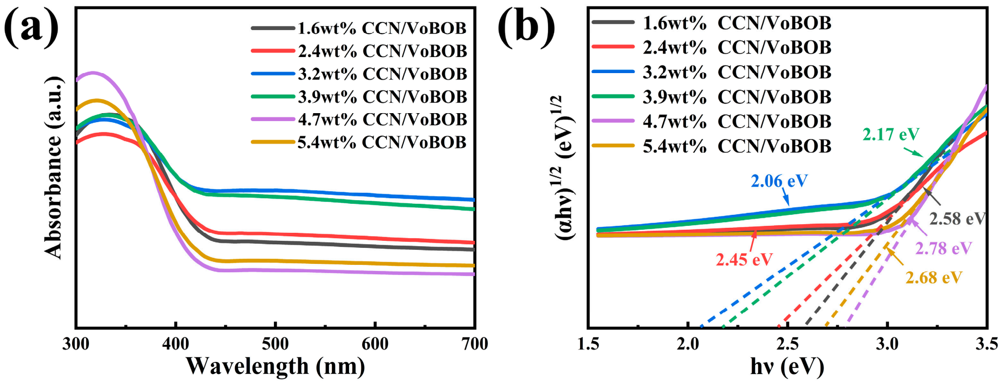

Figure S2. (a) UV-vis DRS, and (b) Tauc plots of 1.6wt% CCN/VoBOB, 2.4wt% CCN/V$_o$BOB, 3.2wt% CCN/V$_o$BOB, 3.9wt% CCN/V$_o$BOB, 4.7wt% CCN/V$_o$BOB and 5.4wt% CCN/V$_o$BOB.

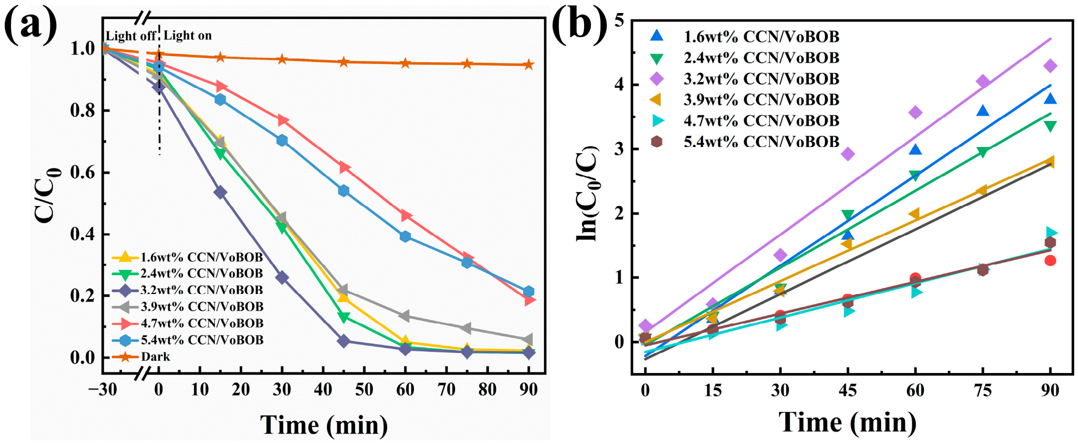

Figure S3. (a,b) Photocatalytic degradation activity of RhB under visible light irradiation for 1.6wt% CCN/V_oBOB, 2.4wt% CCN/V_oBOB, 3.2wt% CCN/V_oBOB, 3.9wt% CCN/V_oBOB, 4.7wt% CCN/V_oBOB and 5.4wt% CCN/V_oBOB.

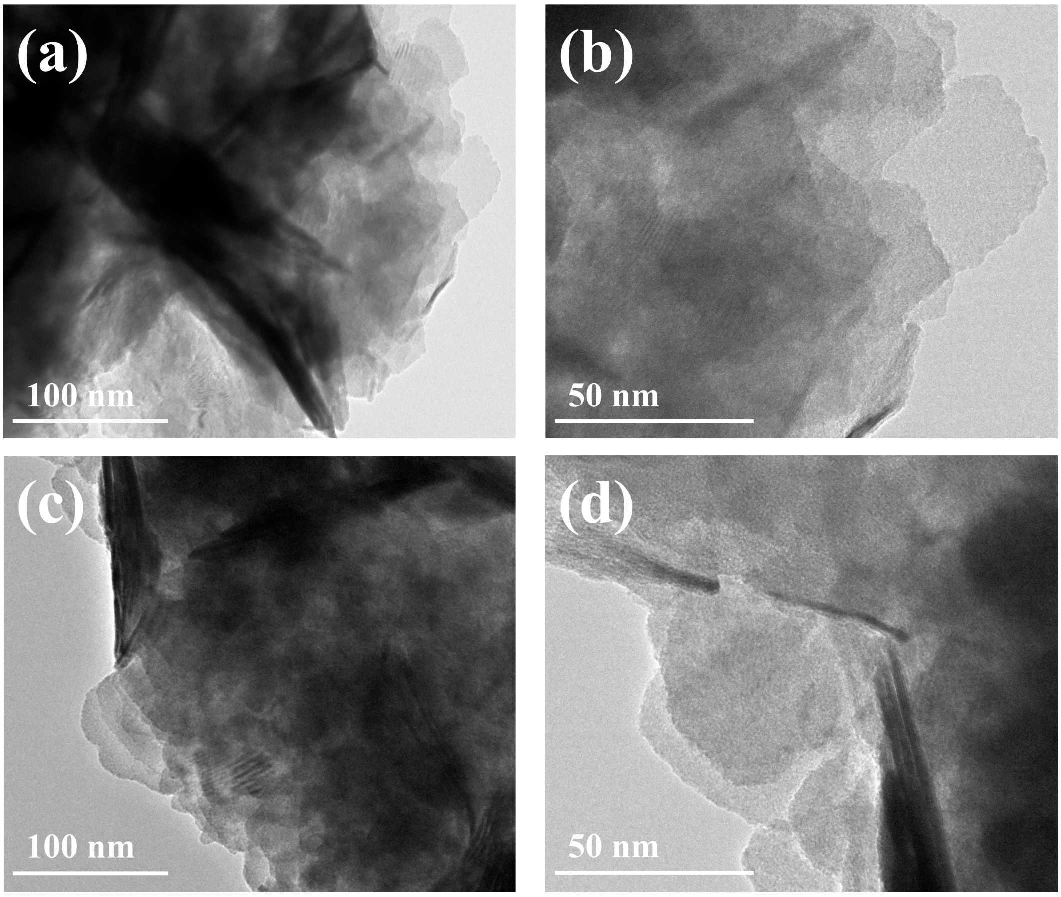

Figure S4. TEM images of the 3.2 wt% CCN/V_oBOB composite (a,b) before and (c,d) after five cycling runs for RhB degradation.

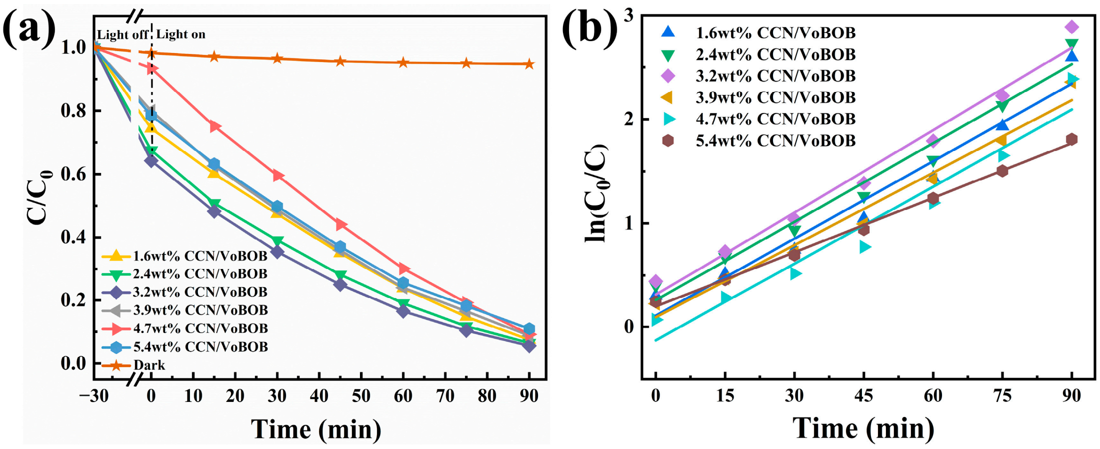

Figure S5. (a,b) Photocatalytic degradation activity of MB under visible light irradiation for 1.6wt% CCN/V_oBOB, 2.4wt% CCN/V_oBOB, 3.2wt% CCN/V_oBOB, 3.9wt% CCN/V_oBOB, 4.7wt% CCN/V_oBOB and 5.4wt% CCN/V_oBOB.

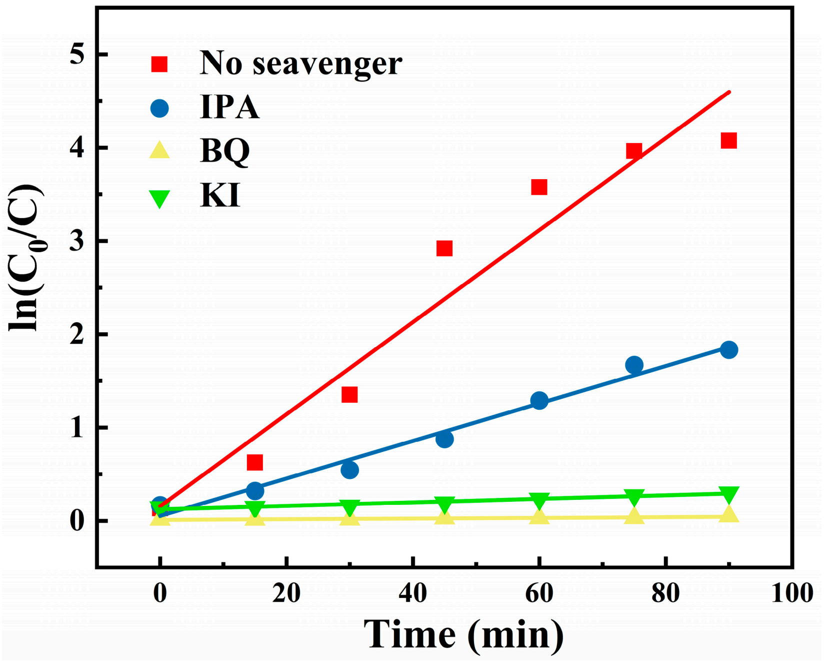

Figure S6. Photocatalytic degradation kinetics of RhB over 3.2 wt% CCN/V_oBOB in the presence of different scavengers.

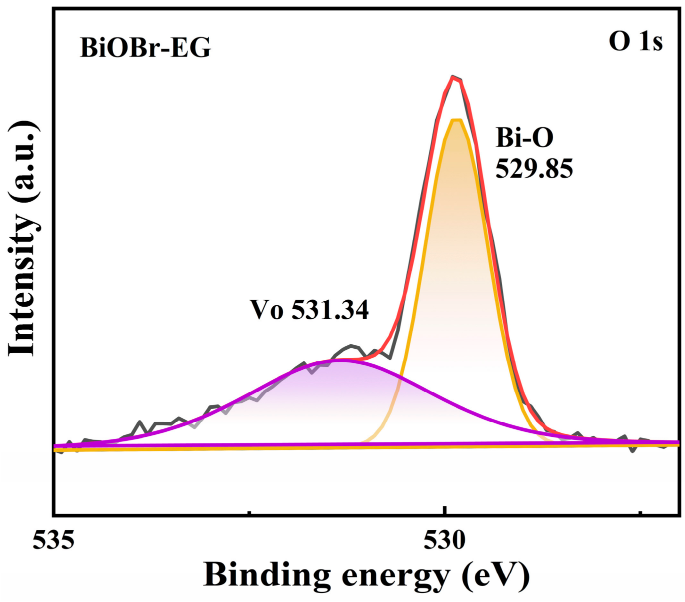

Figure S7. The O 1s XPS spectrum of V_oBOB synthesized without PVP.

Table S1 Surface elemental compositions of CN, CCN, BOB, V_oBOB, and 3.2 wt% CCN/V_oBOB determined by XPS analysis.

**Sample**	**C(at%)**	**N(at%)**	**Bi(at%)**	**Br(at%)**	**O(at%)**
CN	43.89	56.11	-	-	-
CCN	51.42	48.58	-	-	-
BOB	-	-	9.63	11.25	22.66
V_oBOB	-	-	10.76	12.56	23.83
CCN/V_oBOB	43.85	7.08	11.99	13.56	23.51
